# Participation in the house-ball community as a social determinant of HIV care and prevention outcomes among transgender women of color

**DOI:** 10.1016/j.socscimed.2026.119035

**Published:** 2026-01-25

**Authors:** Alexander Furuya, Cho-Hee Shrader, Makella Coudray, Ichiro Kawachi, Asa Radix, Jenesis Merriman, Denton Callander, Dustin T. Duncan

**Affiliations:** a Department of Epidemiology, Columbia University Mailman School of Public Health, New York, NY, USA; b Division of Nursing Science, Rutgers University School of Nursing, Newark, NJ, USA; c Department of Population Health Sciences, College of Medicine, University of Central Florida, Orlando, FL, USA; d Harvard School of Public Health, Boston, MA, USA; e Callen-Lorde Community Health Center, New York, NY, USA; f Kirby Institute, University of New South Wales, Sydney, Australia

**Keywords:** Transgender health, House-ball, HIV, Health equity, Racism, Community health

## Abstract

House-ball scenes around the world have historically been a community for queer people to form chosen families and social networks and be exposed to community health campaigns. We investigated the impact of house-ball participation on HIV care and prevention outcomes among transgender women of color (TWOC) living in New York City. We asked 178 participants who identified as TWOC about their participation in the house-ball scene (never, former, current), and we asked them about their status-neutral HIV care and prevention outcomes, including HIV/STI testing, condom use, PrEP use, and HIV viral load suppression. We used Targeted Maximum Likelihood Estimation (TMLE) to estimate the adjusted relative risk of house-ball participation on these baseline outcomes; we included age, education, and US-born nativity as potential confounders. Among TWOC living with HIV (n = 94; 52.8 %), we found a positive effect of current participation in the house-ball scene on past six-month STI testing compared to never participation (Relative Risk: 1.20, 95 % CI: [1.07, 1.34]). Among TWOC not living with HIV (n = 84; 45.2 %), we found a positive effect of current participation in the house-ball scene on past six-month HIV testing (Relative Risk: 1.27, 95 % CI: [1.11, 1.44]). We found that those who formerly participated in the house-ball scene were less likely to currently use PrEP compared to those who never participated (Relative Risk: 0.44, 95 % CI: [0.23, 0.88]). Community, network connections, and exposure to health campaigns within the house-ball scene may enable and motivate individuals to achieve better HIV care and prevention outcomes.

## 1. Introduction

Transgender women of color are disproportionately burdened by HIV and face barriers to accessing and using prevention and treatment services (Centers for Disease Control and Prevention, 2021; Rebchook et al., 2017). According to a 2024 UNAIDS report, the global median HIV prevalence among transgender individuals is estimated to be 9.2 %, and transgender women have 20 times the risk of acquiring HIV compared to the general population (Joint United Nations Programme on HIV/AIDS, 2024). Based on theories of intersectionality, this may be due to their unique positionality in society and vulnerability to racism, transphobia, and classism (Crenshaw, 1991; Wesp et al., 2019). Further, transgender women of color face structural discrimination in nearly all aspects of life, including housing, education, employment, health, and incarceration (Garcia and Crosby, 2020), all of which could lead to adverse HIV outcomes through pathways mediated through economic deprivation and stress (Borkowski and Borkowska, 2024; Menza et al., 2021). As a result, in the United States, the prevalence of HIV among Black transgender women is 62 %, and the prevalence among Latina transgender women is 35 % (Centers for Disease Control and Prevention, 2021).

In this context of adversity, community resilience may buffer the effects of discrimination and improve HIV care and prevention outcomes among transgender women of color. One definition of community resilience is the ability of communities to “bounce back” from adversity and can be considered as a dynamic process of adaptation (Hall et al., 2023). While the onus of accountability should be placed on structural sources of oppression, community resilience can provide important solace for marginalized individuals (McKeown et al., 2022). An important component of community resilience is social capital, which is defined as the resources (e.g., tangible assistance, emotional support) accessed by members of a community through their social networks (Liu et al., 2022). Social capital can enable good health in several ways. First, health behaviors (and health-relevant information) can “spread” via members of the community through social norms and accepted attitudes (Nieminen et al., 2013). In accordance with the theory of homophily, defined as the tendency for connections to form among those who are alike, health behaviors tend to spread faster among those of similar attributes such as race, gender, age, and cultural practices (Flatt et al., 2012; Shrader et al., 2024). Second, members of the community can share or redistribute tangible, material resources or information in a process called reciprocity (Eriksson, 2011). All of these mechanisms can protect individuals from adverse forces that could otherwise cause poor health (Kawachi, 1999).

A potential source of community resilience for transgender women of color in the United States is the house-ball scene (Kubicek et al., 2013). The house-ball scene consists primarily of transgender, gay, lesbian, bisexual, and queer people of color and has had a long history since its inception in New York City during the Harlem Renaissance in the 1920s (Arnold and Bailey, 2009). House-ball communities can now be found in cities all around the world, including Mexico City, Hong Kong, and London (Villegas, 2016; Liu, 2025; Rasmussen, 2018). While the house-ball scene is famous for the competitions between houses in events called “balls,” in which members compete in specific categories like vogue performance, body, and face, for prizes, including monetary amounts, the house-ball scene can also offer a safe space for otherwise marginalized individuals to congregate and celebrate their identities (Rowan et al., 2013). Members of this community are often part of “houses,” which are social structures that resemble chosen families-complete with house “mothers” and “fathers.” According to Telander et al., house mothers and fathers provide social and material support to their “children,” members of the house, and individuals who may be estranged from or rejected by their biological families due to homophobia and transphobia (Telander et al., 2017). Social connections need not be restrained within houses as members of the overall house-ball scene often “kiki,” or attend less formal balls or other social events with other house families (Telander et al., 2017). Transgender women of color could benefit from these social connections in building social capital that could confer positive health impacts.

Since the early days of the HIV/AIDS pandemic, the New York City house-ball scene has played an active role in combating the spread of HIV. This sustained engagement is rooted in both the high prevalence of HIV within the community and the strengths of its internal social networks. According to Phillips II et al., the house-ball scene was ravaged by HIV, and many members of the community died from AIDS-related complications during the 1980s; as a response, the community developed ways to share resources, such as information about prevention and testing, to prevent the spread of HIV (Phillips II et al., 2011). Community health organizations including the Men of Color for HIV/AIDS (MOCHA) Center have also historically worked with members of the house-ball scene to ensure access to HIV testing and referrals to treatment to members of the house-ball scene (Alio et al., 2020; King-Sumner, 2017). The effort continues in the form of the New York City-hosted Latex Ball, the largest house-ball event in the world; hosted by the GMHC (formerly Gay Men’s Health Crisis), the house-ball event continues to provide HIV and STI testing and ways to access pre-exposure prophylaxis (PrEP) and non-occupational post-exposure prophylaxis (PEP) (GMHC, 2021). Therefore, transgender women of color who participate in the house-ball may be more likely to be exposed to these health campaigns.

Some researchers have found a positive relationship between participation in the house-ball on health outcomes in the United States; however, many of the studies used data collected primarily from men who have sex with men (Kubicek et al., 2013; Murrill et al., 2008; Smith et al., 2023; Young et al., 2017). Transgender women of color may not necessarily have the same experience as men who have sex with men in terms of house-ball participation. Thus, our goal is to understand whether participation in the house-ball scene is associated with HIV care and prevention outcomes among transgender women of color. We hypothesize that participation in the house-ball scene can lead to improved HIV care and prevention outcomes among transgender women of color living in New York City.

## 2. Methods

### 2.1. Study population

We drew data from participants enrolled in the Trying to Understand Neighborhoods and Networks Among Transgender Women of Color (TURNNT) Cohort Study. We recruited participants using social media marketing, print advertising, event-based recruitment, referrals from organizational outreach and partnership, and snowball sampling via referrals from enrolled participants. The research team recruited participants from August 31, 2020, to November 4, 2022. Eligible participants (1) identified as transgender women of color, (2) were between 18 and 55 years old at enrollment, (3) lived in the New York City metropolitan area (including northern New Jersey, Long Island, and the lower Hudson Valley), and (4) spoke either English or Spanish at enrollment. The study sample comprises both individuals living with and without HIV (Furuya et al., 2024).

Data collection occurred in three waves: at enrollment (wave 1), six-month follow-up, and twelve-month follow-up. For this study, we used data from the 6-month follow-up and the 12-month follow-up. The study team administered surveys to the participants in person prior to the COVID-19 lockdown and conducted surveys over Zoom during the lockdown. Participants were compensated with pre-paid gift cards as follows: $50 per interview at wave 1, $75 per interview at wave 2, and $100 per interview at wave 3. Additional details of the TURNNT study can be found elsewhere (Duncan et al., 2024).

We used Duncan’s (2025) Health Equity Research Production Model to structure our study using a framework of accountability (Duncan, 2025). As such, we (1) engaged and centered community leaders from the transgender women of color community and service organizations that support transgender health in designing the TURNNT study, and (2) ensured that identities of the research team reflected our participants’ identities. From these collaborative efforts, individuals from the transgender women of color community and our study team developed a comprehensive survey together. More specifically, we engaged community members in developing the measurements used for the present study. Finally, we shared the manuscript with all of the members of the research team to aid in the interpretation of the results.

### 2.2. Ethics statement

At the initial enrollment interview, a bilingual study team member reviewed the consent form describing the study background, requirements, timeline, confidentiality procedures, and participant rights with interested participants. Participants provided voluntary written consent, then were asked to sign an optional release of information form to allow for the sharing of sexually transmitted infection (STI) and HIV testing information with the study team. The study was approved by the Institutional Review Board at Columbia University Irving Medical Center (IRB-AAAS8164).

### 2.3. House-ball participation

At six-month follow-up, we assessed participation in the house-ball scene by asking participants, “*Have you ever been a member of or participant in the house-ball scene, sometimes known as the ballroom community?*” Participants could respond with “No, never”, “Yes, but I have not participated in the house-ball scene in the past 6 months”, or “Yes, I have participated in the house-ball scene in the past 6 months”. For this analysis, we treated house-ball scene participation as a three-level variable and categorized participants as having never participated in the house-ball scene (reference group), having formerly participated in the house-ball scene, or those currently participating in the house-ball scene. We chose to treat house-ball participation in this way as we believe that each level of participation reflects different social network structures and exposure intensities; for example, those currently participating in the house-ball scene may be more exposed to the social connections and the public health interventions offered these balls, and those who were formerly in the house-ball scene may have different social networks than those currently in it (Green and Gibbs, 2024).

### 2.4. HIV serostatus and STI testing

HIV serostatus was self-reported at the baseline interview as positive, negative, or unknown. Participants who reported a negative or unknown HIV serostatus completed a point-of-care OraQuick saliva HIV test at the second interview of each wave. Participants were mailed the in-home HIV self-test between the first and second interviews at each wave and completed the test over a video call with an interviewer. All participants with a positive test result were immediately referred to confirmatory blood testing with their primary care provider or to a TURNNT partner organization, through a warm handoff. For all other STI measurements, we used self-reported data.

### 2.5. Consistent condom use and HIV prevention and care cascades

At the 12-month follow-up, we ascertained consistent condom use and three HIV prevention and care cascades. For consistent condom use, we asked participants if they always used condoms during penetrative sex in the past 6 months (referent) or if they did not. We assessed the following HIV prevention and care cascades: HIV testing, PrEP use, and HIV viral load suppression. For HIV testing, we asked the subset of those not living with HIV if they had obtained an HIV test in the past year (ref = no test). For current PrEP use, we asked those who were not living with HIV and sexually active if they were currently taking PrEP (ref = not taking PrEP). Finally, for HIV viral load suppression, we asked those living with HIV whether they currently had a detectable or undetectable (<200 virus copies/mL) HIV viral load according to their most recent viral load test (ref = undetectable viral load).

### 2.6. Potential confounders

We measured sociodemographic characteristics of our study population at the baseline survey. Data were collected on baseline age (ref = 18–24 years, 25–34 years, 35–44 years, and 45–55 years), education (ref = high school, less than high school, more than high school), and US-nativity (ref = US-born, or foreign-born). Based on the limited literature on determinants of house ball scene participation, we believe there is some evidence to suggest that sociodemographic and socioeconomic characteristics are associated with house-ball participation (Arnold and Bailey, 2009). These factors are also known to be associated with HIV care and prevention outcomes (Menza et al., 2021). Therefore, we adjusted for age, education, and US-nativity to achieve conditional exchangeability.

### 2.7. Statistical analysis

We performed descriptive analyses to characterize the sociodemographic composition of the study sample and the distribution of the exposure and outcome. We conducted chi-squared tests to assess whether age, education, US-nativity, income, and HIV status were correlates of ever participating in the house-ball scene. We used a doubly-robust method, Targeted Maximum Likelihood Estimation (TMLE), to estimate the adjusted risk ratio that reflects the effect of the house-ball scene participation on HIV care and prevention outcomes, stratified by HIV serostatus, and adjusting for potential confounders. While we assume that accounting for our measured confounders –age, education, and US-nativity– will achieve conditional exchangeability between those exposed and unexposed, we are uncertain of the functional relationships between our variables. We could risk having model misspecification, which could lead to biased estimates of the average effect (Li and Shen, 2020). When we use doubly-robust methods, we create both the propensity model, which models the conditional probability of participating in the house-ball scene given covariates, and the outcome model, which models the conditional treatment effect between participation in the house-ball scene and HIV care and prevention outcomes given covariates. Doubly-robust methods combine information from both the propensity model and the outcome model to calculate the statistical estimand of interest (Funk et al., 2011). The benefit of using this method is that we are able to calculate a minimally biased estimate of the association, even if one of our models is misspecified (Luque-Fernandez et al., 2018). Furthermore, TMLE is suited for machine learning approaches and can therefore incorporate super learner algorithms to further reduce bias arising from model misspecification (Luque-Fernandez et al., 2018); this means that, through machine learning, we can find the optimal functional relationships between the variables.

To conduct TMLE analysis, we used the “LTMLE” package in R (Lendle et al., 2017). The analytical dataset has missing data; however, for any given analysis and outcome, at most only 5 % of observations have to be removed due to missingness. Due to this, we took a complete case approach for our analyses, and we believe that bias due to missingness would be minimal (Dong and Peng, 2013). In a separate analysis, we found that those with missing data and those with complete data were comparable in terms of measured covariates. Our candidate estimators came from the SuperLearner package and included “SL.glm”, “SL. mean”, “SL.bayesglm”, and “SL.earth”. When we conduct TMLE, the algorithm chooses the best weighted combination of candidate estimators through a cross-validation (Lendle et al., 2017). For both the propensity models and outcome models, we included potential confounders –age, education, and US-nativity. All quantitative analyses were conducted using R/R Studio (Version, 2024.09.1, +394).

## 3. Results

In total, 178 participants were included in this analysis. We found that 94 were living with HIV (52.8 %) and 84 were not living with HIV (47.2 %). Table 1 details the characteristics of the study population: The largest age group was between ages 35–44 years (37.6 %); of participants, 33.1 % reported less than a high school education, more than half (52.8 %) had an annual income of less than $10,000, and 58.4 % were US-born. Overall, 41.6 % of those sexually active consistently used condoms during sex in the past 6 months, and 83.1 % of participants took an STI test within the past 6 months. In terms of HIV prevention methods among people not living with HIV, 83.3 % reported having taken an HIV test in the past year, and 42.9 % were currently using PrEP. Among people with HIV (PWH), 95.7 % reported having an undetectable viral load. We found that 6.7 % were currently participating in the house-ball scene, 20.8 % formerly participated in the scene, and 71.9 % never participated. When conducting chi-squared tests, we found that being born in the US was predictive of ever participating in the house-ball scene; those who were born in the US were 4.15 times more likely to ever participate in the house-ball scene compared to those not born in the US. Age, education, income, and HIV status were not found to be correlates of house-ball participation.

Among PWH, we found that current participation in the house-ball scene, relative to never participating in the house-ball scene, was significantly and positively associated with STI testing in the past six months (Relative Risk: 1.20, 95 % CI: [1.07, 1.34]). Among people not living with HIV, we found that current participation in the house-ball scene was significantly and positively associated with HIV testing in the past six months (Relative Risk: 1.27, 95 % CI: [1.11, 1.44). Similarly, former participation in the house-ball scene was positively associated with HIV testing (Relative Risk: 1.24, 95 % CI: [1.10, 1.40) but negatively associated with PrEP use (Relative Risk: 0.44, 95 % CI: [0.23, 0.88]) The effect of house-ball participation on other outcomes was not statistically significant; the full results are displayed in Table 2. We note that the confidence intervals for several of the effects are wide, and this may be due to the sparse cell counts; for example, only 12 individuals were current house-ball participants.

## 4. Discussion

Research about the determinants of health among transgender women of color is paramount for developing interventions that aim to reduce health inequities. Transgender individuals have historically been made invisible in public health research, so research that uses data from transgender women of color is valuable in shedding light on important health insights (Bowleg, 2012). This is true now more than ever, as transgender communities and other marginalized communities are increasingly affected by shifts in federal policy environments (Tordoff et al., 2025). Using doubly-robust methods, we found that current participation in the house-ball scene was predictive of STI testing among transgender women of color with HIV and predictive of HIV testing among transgender women of color not with HIV. Interestingly, we found that former participation in the house-ball scene was predictive of not using PrEP. Overall, we believe that the study adds to the body of literature around the impact of participation in the house-ball scene on HIV care and prevention outcomes among transgender women of color. In this study, we recognize and commend the efforts of the house-ball scene and community health organizations in building resilience among queer people of color. We advocate for this continued partnership and for potential scale-up in HIV care and prevention programming.

Our results about the effect on HIV and STI testing align with those of Arnold et al., who found that social networks and social support among those in the house-ball scene were significantly associated with HIV testing and safer sex practices among house-ball-attending Black men who have sex with men and transgender women (Arnold et al., 2018). Although Khanna et al. found that PrEP awareness was associated with house-ball scene participation among Black men who have sex with men, they did not explore actual PrEP use (Khanna et al., 2016); we found that those who formerly participated in the house-ball scene were less likely to use PrEP compared to those who never participated in the house-ball scene. We suspect that this may be due to the fact that a factor associated with dropout from the house-ball scene may also be associated with PrEP non-use; for example, negative experiences like violence or economic deprivation could cause individuals to drop out of the house-ball scene and also deter them from PrEP use. We did not find a relationship between participation in the house-ball scene and condom use and HIV viral load suppression among TWOC. This suggests that while HIV and STI testing, once-off behaviors, may be disseminated in the house ball scene, more complex behaviors such as condom use and antiretroviral use may not be as “simple” behaviors and require more robust and complex social reinforcement. Further, while HIV and STI testing may be performed during house-ball events, condom and antiretroviral use are behaviors conducted privately.

We believe our results to be robust, given the plausible mechanisms by which participation in the house-ball scene leads to better HIV care and prevention outcomes. First, the structure of the house-ball scene is conducive to the promotion and propagation of healthy behaviors. Arnold & Bailey conducted an ethnographic study examining community practice around HIV prevention and treatment in the house-ball scene; they found that the family structures and dense social networks of the house-ball scene enables individuals to practice HIV prevention and treatment behaviors; more specifically, nurturing house mothers and fathers as guides acted as role models who can guide their house children to practice safe behaviors, such as consistent condom use (Arnold and Bailey, 2009). We can also understand our results using network effect theory; because those in the house-ball scene are part of dense social networks, the diffusion of healthy behaviors, including HIV care and prevention practices, tends to be faster and concentrated (Latkin and Knowlton, 2015; Shrader et al., 2024). Second, the direct cooperation of the house-ball scene and community health organizations may have led to better HIV care and prevention outcomes (Phillips II et al., 2011). According to Alio et al., house leaders are aware of HIV educational activities led by community-based organizations in the house-ball scene; some house leaders even addressed HIV during their house meetings and highlighted several of these community resources (Alio et al., 2020). To get a better sense of how house-ball participation impacts HIV care and prevention outcomes, we invite other researchers to conduct contemporary studies examining how participation in the house-ball scene affects health; researchers could utilize qualitative methods or a robust social network to understand this relationship.

There are several limitations in this study. First, we assumed that including age, education, and US-born nativity as our confounder set was sufficient to achieve conditional exchangeability. However, it is possible that there is unmeasured confounding, such as stigma, discrimination, and HIV risk perception, that could have biased our results. Second, the measurement and operationalization of the exposure may be overly simplistic and therefore prone to measurement error. In our study, we assume that the effect of participation in the house-ball scene is well-defined; however, this may not be the case, as people who participate in the house-ball community often have diverse experiences (Alio et al., 2020). Additionally, our question may exclude those who participate in informal house systems or those who do not belong to an established house but are still associated with the scene. Third, there may be measurement error in HIV care and prevention outcomes because we relied on self-reported data. From the literature, it appears that self-reported HIV care and prevention outcomes are accurate, but there may be potential misreporting due to social desirability (Povshedna et al., 2025; Qasmieh et al., 2022). Finally, some of the analyses may be more vulnerable to random error due to small cell sizes. For example, only 12 participants reported current house-ball participation, and only 4 participants reported detectable viral load. Furthermore, we note that in our study, the confidence intervals estimated for these effects were relatively wide, indicating that the sample size for the analysis may have been too small to detect a meaningful effect. However, we also note several strengths of this study. Because we used TMLE, we are less likely to have issues of model misspecification compared to traditional regression methods. This is because TMLE is a doubly-robust method that uses machine learning to find the optimal functional relationship between the variables. Additionally, the TURNNT cohort is one of the largest study samples of transgender women of color, and therefore, we believe that the findings from this cohort have high internal validity. In the future, we hope to replicate these findings in a larger sample to understand the potential effect of house-ball participation on PrEP use.

In terms of external validity, we note that the results from the TURNNT cohort may not necessarily be generalizable to all transgender women of color in New York City given our non-probability method of sampling. However, we believe that our method of recruitment, while it may limit generalizability, could increase relevance for intervention development for those in the community; interventions informed by our findings would be relevant for populations similar to TURNNT.

In conclusion, we found that participation in the New York house-ball scene improved some HIV care and prevention outcomes among transgender women of color. The New York house-ball scene has a rich history that is characterized by a flourishing community of LGBTQ + people of color, where diverse, intersectional identities were celebrated. While the house-ball scene studied in this paper is specific to the context of the United States, we believe that our findings could apply to similar communities around the world. In fact, the house-ball scene has a global presence extending to Europe, Latin America, and Asia, and could have similar health benefits to marginalized individuals around the world (Plaster, 2022). Through communal social support, individuals of the ballroom scene could motivate each other to practice healthy behaviors and connect to health resources (Arnold and Bailey, 2009). We see this as a form of community resilience; individuals can find social capital in this scene that enables them to be healthier. Future research can consider using a qualitative study or a robust longitudinal social network study to characterize how HIV care and prevention strategies work in the context of the house-ball scene. Based on this work, we encourage other researchers to conduct strength-based research and to identify sources of community resilience. By identifying these leverage points, we can bolster community resilience and reduce health inequities experienced by marginalized populations.

## Figures and Tables

**Table 1:** Characteristics of the study population, the TURNNT cohort study.

	Proportion (n = 178)

Race and Ethnicity	
Black	23.6 %
Latina	47.8 %
Multiracial/Biracial	23.6 %
Asian/Asian American	2.8 %
Middle Eastern/North African	1.1 %
Another Race or Ethnicity	1.1 %
Age	
18 to 24	7.3 %
24 to 34	26.4 %
35 to 44	37.6 %
45 to 55	27.0 %
Missing	1.7 %
Education	
Less than High School	33.1 %
High School	28.1 %
More than High School	38.8 %
US-Born	
Yes	58.4 %
No	41.0 %
Missing	0.6 %
Income	
No Income	12.9 %
$1 to $9999	39.9 %
$10,000 to $29,999	23.0 %
$ 30,000 to $49,999	7.3 %
$50,000 or more	6.7 %
Missing	10.1 %
Ball Room Participation	
Yes, currently participating	6.7 %
Yes, but no longer participating	20.8 %
No, never	71.9 %
Missing	0.6 %
HIV Status	
Positive	52.8 %
Negative	47.2 %
Past 6 Month STI (non-HIV) Testing	
Yes	83.1 %
No	16.9 %
Always Condom Use	
Yes	32.0 %
No	44.9 %
Missing or Not Having Sex	23.1 %
Past 6 Month HIV Testing^a^	
Yes	83.3 %
No	16.7 %
Current Pre-Exposure Prophylaxis Use^a^	
Yes	42.9 %
No	57.1 %
Current Undetectable Viral Load^b^	
Yes	95.7 %
No	4.3 %

a Among people not living with HIV.

b Among people living with HIV.

**Table 2:** Effect of House-Ball Participation on HIV Care and Prevention Outcomes Using Doubly-Robust Methods. Using doubly-robust estimators, we estimated the effect of participation in the house-ball scene on HIV care and prevention outcomes (condom use, HIV/STI testing, undetectable viral load, and PrEP use), adjusting for age, education, and US-nativity.

A. Among People Living with HIV				

		Always Condom Use	Past 6 Months STI Testing	Undetectable Viral Load

Not Currently in the House-ball Scene	Ref.	Ref.	Ref.	Ref.
Formerly in the House-Ball Scene	Adjusted Risk Ratio [95 % Confidence Interval] Analytical Sample Size	0.89 [0.55, 1.43] n = 61	0.94 [0.76, 1.17] n = 86	0.94 [0.81, 1.09] n = 86
Currently in the House-ball Scene	Adjusted Risk Ratio [95 % Confidence Interval] Analytical Sample Size	1.15 [0.52, 2.54] n = 49	**1.20 [1.07, 1.34] n=68**	1.03 [0.99, 1.08] n = 68

B. Among People Not Living with HIV					

		Always Condom Use	Past 6 Months STI Testing	Current PrEP Use	Past 6 Months HIV Testing

Not Currently in the House-ball Scene	Ref.	Ref.	Ref.	Ref.	Ref.
Formerly in the House-Ball Scene	Adjusted Risk Ratio [95 % Confidence Interval Analytical Sample Size	0.93 [0.45, 1.91] n = 62	1.13 [0.99, 1.29] n = 75	**0.44 [0.23, 0.88] n=75**	**1.24 [1.10, 1.40] n=75**
Currently in the House-ball Scene	Adjusted Risk Ratio [95 % Confidence Interval] Analytical Sample Size	0.58 [0.29, 1.18] n = 60	0.78 [0.51, 1.20] n = 70	0.78 [0.37, 1.61] n = 70	**1.27 [1.11, 1.44] n=70**

## Data Availability

The data that support the findings of this study are available from the corresponding author, upon reasonable request.

## Data availability

Data will be made available on request.
